# Planting Geometry May Be Used to Optimize Plant Density and Yields without Changing Yield Potential per Plant in Sweet Corn

**DOI:** 10.3390/plants13172465

**Published:** 2024-09-03

**Authors:** Atom Atanasio Ladu Stansluos, Ali Öztürk, Aras Türkoğlu, Magdalena Piekutowska, Gniewko Niedbała

**Affiliations:** 1 Department of Field Crops, Faculty of Agriculture, Upper Nile University, Malakal 71100, South Sudan; atomtaban@gmail.com; 2 Department of Field Crops, Faculty of Agriculture, Ataturk University, 25240 Erzurum, Türkiye; 3 Department of Field Crops, Faculty of Agriculture, Necmettin Erbakan University, 42310 Konya, Türkiye; 4 Department of Botany and Nature Protection, Institute of Biology, Pomeranian University in Słupsk, 22b Arciszewskiego St., 76-200 Słupsk, Poland; magdalena.piekutowska@upsl.edu.pl; 5 Department of Biosystems Engineering, Faculty of Environmental and Mechanical Engineering, Poznań University of Life Sciences, Wojska Polskiego 50, 60-627 Poznań, Poland

**Keywords:** *Zea mays* L. *saccharata*, plant population, plant growth, LAI, kernel yield

## Abstract

Planting geometry is one of the most important management practices that determine plant growth and yield of corn. The effects of eight planting geometries (35 × 23 cm, 40 × 21 cm, 45 × 19 cm, 50 × 18 cm, 55 × 17 cm, 60 × 16 cm, 65 × 15 cm, 70 × 15 cm) on plant growth and yields of three sweet corn hybrids (Argos F_1_, Challenger F_1_, Khan F_1_) were investigated under Erzurum, Türkiye conditions in 2022 and 2023 years. Variance analysis of the main factors shows a highly significant effect on whole traits but in two-way interactions some of the traits were significant and in the three-way interactions, it was insignificant. As an average of years, the number of plants per hectare at the harvest varied between 92,307 (35 × 23 cm) and 120,444 (70 × 15 cm) according to the planting geometries. The highest marketable ear number per hectare (107,456), marketable ear yield (24,887 kg ha^−1^), and fresh kernel yield (19,493 kg ha^−1^) were obtained from the 40 × 21 cm planting geometry. The results showed that the variety Khan F_1_ grown at 40 × 21 cm planting geometry obtained the highest marketable ear number (112,472), marketable ear yield (29,788 kg ha^−1^), and fresh kernel yield (22,432 kg ha^−1^). The plant density was positively correlated with marketable ear number (r = 0.904 **), marketable ear yield (r = 0.853 **), and fresh kernel yield (r = 0.801 **). The differences among the varieties were significant for the studied traits, except for plant density and kernel number per ear. In conclusion, the variety Khan F_1_ should be grown at the 40 × 21 cm planting geometry to maximize yields under study area conditions without water and nutrient limitations.

## 1. Introduction

Maize (*Zea mays* L.), including sweet corn, is vital for global food security. Sweet corn, with its high sugar content, is gaining economic importance worldwide. Around 1,105,213 hectares of sweet corn are cultivated globally density [1,2] with the United States leading production [3]. Sweet corn ears are harvested when the kernel moisture content is about 70%, offering fresh, canned, or frozen options for year-round consumption. Sweet corn is prized for its flavor and high nutritional value, rich in essential nutrients like copper, potassium, total soluble solids, lactose, total sugars including sucrose, and vitamins A, C, B3 (niacin), and B9 (folic acid) [4,5]. In Türkiye, sweet corn occupies a small portion of the total corn cultivation area but is economically significant. Türkiye ranks corn as its third most important crop. Compared to 2000, global corn cultivation area, yield, and production of corn increased by 48.6%, 32.3%, and 96.5%, respectively; in Türkiye, it increased by 64.9%, 124.1%, and 269.6%, respectively [6,7].

Maximizing maize production becomes essential when arable land decreases and urbanization rises [8]. Adopting density-tolerant maize varieties and applying scientific field management techniques may significantly improve grain yields [9]. Increasing planting density is an effective strategy for optimizing maize yields [10]. With the increase of the plant density, the leaf area index (LAI) increases especially when there is optimal distribution and arrangement of the plant, and accordingly, the plant canopy will increase their productivity, source–sink accumulation, and growth and kernel yield [11,12,13]. With current hybrids displaying genetic tolerance to higher densities, optimal plant density has increased over time from 30,000 plants ha^−1^ in the 1930s [2,14], reaching 139,000 plants ha^−1^ [15]. On the other hand, reduced carbon and nitrogen uptake, resource competition, and lower yields particularly yield per plant can result from excessive plant density [1,2].

Optimal planting geometry is crucial for efficient resource usage and potential yields, as studies have demonstrated that uniform plant distribution over reduced row spacing can improve optimum plant density and yield [15,16]. In order to optimize plant development, and grain and biomass yield, agronomic methods such as efficient spacing together with suitable varieties of maize are necessary. Optimal plant geometry increases dry matter and yield by improving maize growth, sunlight interception, and radiation use efficiency. Developing and implementing sustainable agronomic production techniques becomes essential as input costs rise and environmental concerns intensify [17,18]. Planting densities influence the photosynthetic capacity, leaf growth, and consumption of light on the structure and function of the maize canopy [5,19]. According to Ming et al. [20], higher plant densities can compensate for lower yield per plant, but too much-crowding stress can reduce yield per unit area. To increase maize population production, it is essential to monitor both plant population growth and individual plant development. Whereas low plant density resulted in a high kernel number per ear [21] and reduced the number of fall armyworms [22]. Spacing is an agronomic technique that requires consideration. The ideal distance between and within rows depends on the variety of crops being grown, soil moisture content, and weed infestation level. While the majority of suitable agronomic techniques and maize requirements have been researched and determined, limited knowledge is known about plant population and row arrangement in relation to many factors, such as soil fertility condition, variety height, days to maturity, etc. [23]. A major way to enhance crop production and land productivity is to increase planting density [24,25]. High plant density, however, can reduce the light intensity that reaches the lower canopy, which can have an impact on crop productivity by influencing how nutrients and light resources are used [17]. Planting density can be changed to maximize canopy structure, enhance light distribution, and increase production and light energy utilization [5,26]. Maximizing sweet corn productivity under particular climatic conditions requires determining the ideal plant density and planting geometry [27].

Rising input prices and the negative effects of modern agriculture on environmental quality, necessitate the development of more sustainable production techniques. For more efficient cultivation areas, light, water, and nutrient resources, optimal planting geometry is critical in modern maize cultivation. Despite genetic advances in tolerance to high plant density, the optimum planting geometry for modern maize hybrids has not been thoroughly investigated, considering both inter- and intra-row spacing. Although there is little research on sweet corn planting geometry, the current studies aimed to determine the optimal plant density by examining the effects of eight different planting geometries and three sweet corn varieties for a better understanding of yield and yield components responses and some morphological and physiological characteristics.

## 2. Results

This experimental field study consisted of 2 growing years, 2022 and 2023, with the findings displayed in the tables below, which all represent the combined statistical results of the two years.

### 2.1. Days to Tasseling, Days to Silking, and Days to Tasseling Silking Interval

The results of the analysis of variance for days to tasseling are given in Table 1. It was determined that the effects of the growing year (Y), planting geometry (PG), variety (V), year × planting geometry interaction, and year × variety interaction on days to tasseling were significant whereas variety × planting geometry and year × planting geometry × variety interactions were insignificant (Table 1). According to planting years, geometries, and varieties, days to tasseling varied between 63.8–67.7 days, 64.9–66.6 days, and 64.4–67.7 days, respectively. The shortest days to tasseling were observed in the year 2023, 35 × 23 cm PG, and Argos F_1_ variety. The longest days to tasseling were determined in the year 2022, 55 × 17 cm PG, and Khan F_1_ variety (Table 2).

The analysis of variance revealed a noteworthy interaction effect between the cropping year and both the planting geometry and varieties, indicating that the cropping year significantly influences the selection of planting geometry and varieties. The statistical analysis indicated significant interactions between cropping year and both planting density as well as variety. However, the overall interaction involving cropping year, planting density, and variety did not exhibit statistical significance for the traits examined in this study (Table 1).

### 2.2. Plant Height and First Ear Height

The differences between the growing years and the varieties in terms of plant height were found significant, while the effects of planting geometry, year × planting geometry interaction, year × variety interaction, variety × planting geometry interaction, and year × planting geometry × variety interaction were found insignificant (Table 1). Plant height varied between 194.9–203.0 cm according to the growing years, 197.6–200.3 cm according to planting geometries, 194.4–205.6 cm according to varieties, and 186.9–208.6 cm in variety × planting geometry interactions. The tallest plant height was measured in the year 2023, with planting geometry of 50 × 18 cm and 60 × 16 cm, Khan F_1_ variety, and the interaction of Khan F_1_ (35 × 23 cm). It was determined that there were significant differences between the growing years and the varieties in terms of the first ear height (Table 2). The first ear height varied between 53.8–59.2 cm according to the years, 54.6–58.0 cm according to planting geometries, 52.2–61.1 cm according to varieties, and 49.9–62.9 cm for variety × planting geometry interactions. The lowest first ear height was measured in the year 2022, 60 × 16 cm planting geometry, Challenger F_1_ variety, Challenger F_1_ (35 × 23 cm), and Challenger F_1_ (45 × 19 cm) interactions (Table 2).

### 2.3. Plant Density, Leaf Area Index, and Stem Diameter

The results of the variance analysis of the number of plants per hectare at the harvesting date according to the eight-planting geometry of the three sweet corn varieties and the number of plants per hectare based on the treatments are presented in Table 1. The effect of planting geometry on plant density was significant, while the effect of the growing year, variety, year × planting geometry interaction, year × variety interaction, variety × planting geometry interaction, and year × variety× planting geometry interaction was insignificant (Table 1). The number of plants per hectare at the harvesting date varied between 92,307 and 120,444 according to the planting geometries, and the highest and lowest values were determined in the planting geometries of 35 × 23 cm and 70 × 15 cm, respectively (Table 2). The results of the variance analysis of the LAI values and the leaf area indices according to the treatments are shown in Table 1. The effect of the growing year, planting geometry, and variety on the LAI was found significant, while the effect of year × planting geometry interaction, year × variety interaction, variety × planting geometry interaction, and year × variety × planting geometry interaction was found insignificant. The LAI varied between 3.09–3.92 according to growing year, 3.01–4.15 planting geometries, 3.32–3.71 according to varieties, and 2.76–4.64 according to variety × planting geometry interactions. The highest LAI was determined in the year 2023, 35 × 23 cm planting geometry, Khan F_1_ variety, and Khan F_1_ (35 × 23 cm) interaction. The treatments with the lowest LAI were the 2022 growing year, 70 × 15 cm planting geometry, Challenger F_1_ variety, and the Challenger F_1_ (70 × 15 cm) interaction. The effect of the growing years and variety were statistically significant on stem diameter while the effect of planting geometry, year × planting geometry, variety × planting geometry, and year × variety × planting geometry treatments was found statistically insignificant (Table 1). Stem diameter values ranged from 17.7–18.9, 17.8–18.8, 17.9–18.8, and 17.1–19.7 mm according to growing years, planting geometries, varieties, and variety × planting geometry interactions, respectively. The largest stem diameter was measured in the 2023 growing year, 70 × 15 cm planting geometry, Khan F_1_ variety, and the interaction of Khan F_1_ (60 × 16 cm) (Table 2).

### 2.4. SPAD Chlorophyll Value and Maximum Quantum Efficiency of PSII (Fv/Fm)

It was determined that the effect of variety on the chlorophyll value was significant, while the effect of the growing year, planting geometry, variety × planting geometry interaction, and year × variety × planting geometry interaction was not significant (Table 1). Leaf chlorophyll values varied between 54.6–54.8 SPAD units according to the growing years, 54.2–55.8 SPAD units according to planting geometries, 53.6–55.3 SPAD units according to varieties, and 52.1–57.2 according to variety × planting geometry interaction. The highest values were measured in the growing year 2022, 35 × 23 cm planting geometry, Khan F_1_ variety, and Khan F_1_ (50 × 18 cm) while the lowest values were measured in the growing year 2023, 60 × 16 cm planting geometry, Challenger F_1_ variety, and Challenger F_1_ (65 × 15 cm) interaction.

The results of the analysis of variance regarding the effect of the treatments on Fv/Fm values are in Table 2. It was determined that the effect of planting geometry, variety, year × planting geometry interaction, year × variety interaction, and variety × planting geometry interaction on the maximum quantum efficiency of PSII value was significant, while the effect of other treatment factors (growing year and year × variety × planting geometry interaction was insignificant. It was determined that the maximum quantum efficiency of PSII values varied between 0.754 and 0.791 according to planting geometries, 0.773–0.785 varieties, and 0.741–0.800 variety × planting geometry interactions. The highest maximum quantum efficiency of PSII value was obtained in the 50 × 18 cm planting geometry, followed by the 55 × 17 cm planting geometry, Khan F_1_ variety, and Challenger F_1_ (50 × 18 cm) and Khan F_1_ (45 × 19 cm) interactions. The lowest value was measured at a planting geometry of 70 × 15 cm, Argos F_1_, and Challenger F_1_ (60 × 16 cm) interaction (Table 2).

### 2.5. Ear Number per Plant, Ear Yield, Marketable Ear Number, and Marketable Ear Yield

The results of the analysis of variance regarding the effect of applications on the ear number per plant are shown in Table 1. The effect of planting geometry and variety on the ear number per plant was significant, while the effects of the growing year, year × planting geometry interaction, year × variety interaction, variety × planting geometry interaction, and year ×variety × planting geometry were insignificant. The highest ear number per plant was found (1.10) from the growing year 2022, (1.18) from the 50 × 18 cm planting geometry, (1.12) from the Khan F_1_ variety, and (1.20) from the Challenger F_1_ (50 × 18 cm) (Table 2).

Ear yield ranged from 21,287–25,289 kg ha^−1^ according to the growing years, 19,815–26,495 kg ha^−1^ planting geometries, 21,912–25,114 kg ha^−1^ varieties, and 18,875–29,788 kg ha^−1^ variety × planting geometry interactions (Table 2). The lowest yield was obtained from the growing year 2023, 70 × 15 cm, Argos F_1_, and the interaction of Argos F_1_ (70 × 15), and the highest yield was obtained from the growing year 2022, 40 × 21 cm planting geometry, Khan F_1_ variety, and Khan F_1_ (40 × 21 cm) interaction (Table 2). The ear yield of 40 × 21 cm planting geometry (26,495 kg ha^−1^) and Khan F_1_ variety (25,114 kg ha^−1^) was significantly superior to the other PGs and varieties. The responses of sweet corn varieties to the different planting geometries in terms of ear yield were significant. Among the variety × planting geometry interactions, the Khan F_1_ (40 × 21 cm) interaction with 29,788 kg ha^−1^ provided the highest ear yield (Table 2).

The effect of year, planting geometry, and variety on the number of marketable ears were significant, while the effects of year × planting geometry, year × variety, variety × planting geometry, and the year × variety × planting geometry interaction were not significant (Table 1). The number of marketable ears varied between 89,480.7–93,061.0 per hectare according to the growing years, 75,903.4–107,455.9 planting geometries, 88,744.9–95,545.0 varieties, and 72,979.4–112,473.5 according to variety × planting geometry interactions. The highest number of ears was obtained from the growing year 2022, 40 × 21 cm planting geometry, Khan F_1_ variety, and Khan F_1_ (40 × 21 cm) interaction. It has been determined that growing year, planting geometry, and variety effects were significant on marketable ear yield (Table 2). Marketable ear yields varied between 18,871.2–22,833.7 kg ha^−1^, 17,087.3–24,887.2 kg ha^−1^ according to planting geometries, 19,840.7–22,185.9 kg ha^−1^, varieties, and 16,264.4–26,420.3 kg ha^−1^ according to variety × planting geometry interactions. The highest yields were obtained from the year 2022, 40 × 21 cm planting geometry, Khan F_1_ variety, and Khan F_1_ (40 × 21 cm) interaction, while the lowest was determined in the year 2023, 70 × 15 cm PG, Argos F_1_ variety, and the interaction of Argos F_1_ (70 × 15 cm) (Table 2).

### 2.6. Kernel Number per Ear and Fresh Kernel Yield

In this study, except for the year, year × planting geometry, and year × variety interactions, the effects of the treatment factors on the kernel number per ear were not significant (Table 1). It was determined that the kernel number per ear was between 656.3–669.8 kernels according to the years, 649.0–673.9 kernels according to planting geometries, 658.7–668.4 kernels according to varieties, and 627.4–687.8 kernels according to variety × planting geometry interactions. The highest values were obtained from the year 2023, 55 × 17 cm PG, Khan F_1_ variety, and the interaction of Challenger F_1_ (60 × 16 cm), whereas the lowest were obtained from the year 2022, 45 × 19 cm PG, Challenger F_1_ variety, and Challenger F_1_ (45 × 19 cm) interaction. It demonstrated that the differences among the treatment factors in terms of fresh kernel yield were significant, except for interactions of growing year × varieties, planting geometry × varieties, and year × variety × planting geometry interaction (Table 1). Fresh kernel yield varied between 13,975–16,574 kg ha^−1^, 11,387–19,493 kg ha^−1^, and 14,213–17,304 kg ha^−1^, according to the growing years, planting geometries, and varieties, respectively. The highest values were determined from the 2022 growing year, and 40 × 21 cm PG and the lowest was from the year 2023, and 70 × 15 cm PG. The Khan F_1_ variety had the highest fresh kernel yield (17,304 kg ha^−1^), followed by the Argos F_1_ and Challenger F_1_ varieties. Fresh kernel yields varied between 9965–22,432 kg ha^−1^ according to variety × planting geometry interactions, and the interaction of Khan F_1_ (40 × 21 cm) provided the highest yield (Table 2).

### 2.7. Correlations Coefficients Result among Traits

Based on the interaction effect of the traits of this study, the analysis of the correlation reveals a positive and significant correlation between most of the traits (Table 3). The PH showed highly significant and positive correlations with LAI (r = 0.832 **), EY (r = 0.784 **), MEN (r = 0.904 **), MEY (r = 0.853 **), and FKY (r = 0.801 **). The LAI correlated positively and significantly with EY (r = 0.775 **), MEN (r = 0.828 **), MEY (r = 0.748 **), and FKY (r = 0.782 **). Besides that, there were significant and positive correlations between ENP and EY (r = 0.435 *), MEN (r = 0.498 *), MEY (r = 0.501 *), and FKY (r = 0.496 *); also, EY correlated positively and significantly with MEN (r = 0.845 **), MEY (r = 0.920 **), and FKY (r = 0.847 **). In addition, MEN was correlated with MEY (r = 0.929 **) and FKY (r = 0.957 **); MEY correlated significantly with FKY (r = 0.910 **) (Table 3).

## 3. Discussion

Optimal planting geometry is the highest plant density at which genetic potential can be maintained at the plant level, and this goal can be achieved by reducing row spacing and providing more uniform plant distribution over the field [28,29]. The combined results clearly showed that the highest marketable ear number per hectare, marketable ear yield, and fresh kernel yield were obtained from the 40 × 21 cm planting geometry. The difference in high marketable ear number and marketable ear yield and the fresh kernel yield might be due to row spacing, and plant density in the positive correlation with the light intercepted during tasseling [30,31]. Besides that, Bhatt [32] stated that different inter-row spacing and intra-row spacing can influence the ear number per hectare and the fresh ear yield per hectare, and the highest mean values were determined from 40 × 25 cm and 50 × 25 cm, respectively.

The results of this study in terms of plant height have significantly differed among the varieties. Usually, early varieties are shorter while late varieties have taller plant height, which can change according to environmental conditions as well as cultivation techniques such as plant density, fertilization, and sowing date. There are several studies investigated and demonstrated the reason/s why the plant height might significantly differ; some of these reasons are; variety and intra-row spacing [33], Stansluos et al. [34] demonstrated a significant difference in terms of plant height of sweet corn varieties (170.0–216.0 cm); Argos F_1_ (183.5 cm), Challenger F_1_ (186.4 cm), and Khan F_1_ (194.4 cm). Additionally, Porte et al. [29,35] demonstrated an increase in plant height and first ear height. Li et al. [11] demonstrated an increase in inter-row spacing. Moreover, Thakur et al. [36] determined the tallest plant at 30 × 20 cm and the widest stem diameter at 60 × 20 cm planting geometry. The reason behind that increase in plant heights might be the competition for light, which mostly makes the internodes thinner and taller. Not only the genetic structure of the varieties, but also the plant height and the stem diameter can be affected by close inter-row and intra-row spacing (high plant density), high doses of nitrogen fertilizers, climatic conditions, and delayed sowing dates [37,38,39,40].

Khan et al. [41] studied two sweet corn varieties in Pakistan and found that higher plant densities led to delayed tasseling and maturity, along with decreases in kernel number and weight per ear. However, the most significant kernel yield was achieved at a plant density of 100,000 plants ha^−1^.

The interval between days to tasseling and days to silking may influenced by the plant density and distribution through plant microclimate. Optimum planting geometry can ensure the best plant distribution accordingly. Uniform plant growth and development (tasseling and silking). Concerning days to tasseling, days to silking, and the interval the results of our experiment initially, the genetic difference of the varieties might be accounted for, thus other factors such as planting geometry which is a key factor in plant growth, development, and yield components. In previous studies, days to silking varied differently. Thus, in their study, Sönmez et al. [42], days to silking ranged between 69.8- 74.7 days in Türkiye, Ordas et al. [39] 65.6–74.9 days under Spain conditions, and Khan et al. [41] determined it to be between 59.0–62.0 days in Pakistan conditions. In this experiment days to silking was almost in the same range. Days to silking is one of the important traits under ecological conditions with short vegetative periods, such as the one of Erzurum, and optimal planting geometry with appropriate variety can guarantee the maturity and harvest confidently.

Depending on the results of Temesgen and Kebena [33] there was a significant in plant height which was between (235.5–263.3 cm) in terms of variety; while it was insignificant in terms of intra-row spacing (84.1–85.2 cm) and interactions. Biswas et al. [37] found a significant difference in both varieties (200.0–231.7 cm) and spacing (208.0–222.5 cm) in terms of plant height, showing that narrow spacing resulted in shorter plants than wide spacing. Stansluos et al. [34] demonstrated a significant difference in terms of plant height of sweet corn varieties (170.0–216.0 cm); Argos F_1_ (183.5 cm), Challenger F_1_ (186.4 cm), and Khan F_1_ (194.4 cm). Usually, early varieties are shorter while late varieties have taller plant height, which can change according to environmental conditions as well as cultivation techniques such as plant density, fertilization, and sowing date. In the previous research conducted by Ordas et al. [39] and Sönmez et al. [42], sweet corn varieties were significantly varied in terms of plant height. With insignificant differences among the planting geometries, Porte et al. [35] demonstrated an increase in the plant height with the increase of the inter-row spacing from 50 × 20 cm to, 60 × 20 cm followed by 75 × 20 cm. Li et al. [11] stated that plant height and first ear height increased from 267.0 to 304.0 cm and from 116.0 to 138.0 cm, respectively, with the increase of the planting density, demonstrating a significant interaction between plant height and first ear height. Thakur et al. [36] conducted a study examining various planting geometries and nitrogen levels, as well as farm-yard-manure levels. They found that the tallest plants were observed at a planting geometry of 30 × 20 cm, while the widest stem diameter was recorded at a planting geometry of 60 × 20 cm.

In this study, significant effects of the key factors (cropping year, planting geometry, and variety) were determined; the values of LAI in our study is within the ranges of Walia et al. [40] and Maresma et al. [38]. Mostly, the leaf area index is affected by several factors such as the genetic structure (canopy structure) of each genotype [43], planting geometry (intra-row spacing), plant density, planting date, and nitrogen doses. The leaf area index was proved to be increased with the increase of inter-row spacing [33], increase of plant density [26], decrease with delay of planting date [44], and increase of nitrogen doses [36].

Despite the absence of significant differences in planting geometry, Walia et al. [40] observed an increase in LAI from 3.21 to 4.22 with a decrease in intra-row spacing, ranging from 30 × 20 cm to 30 × 10 cm, respectively. In another experiment, Maresma et al. [38] demonstrated that delay in planting date increases LAI from 3.57 in the mid-March planting date to 4.88 in the mid-May planting date. In addition, a linear increase in LAI was determined by Liu et al. [26], which was supported by Li et al. [11], proving that an increase in plant density from 1.5 to 18 plants m^2-1^ leads to an increase of LAI from 1.08 to 10.18. An increase in plant density from 94,000 to 139,000 plants ha^−1^ leads to an increase in the LAI from 5.8 to 7.3 [15]. As plant density and planting geometry nitrogen doses also increase LAI as determined by Thakur et al. [36]. Except for the inter-row spacing, which showed a significant difference, the highest plant (279.1 cm) was determined at 55 cm inter-row spacing, intra-row spacing, and the interactions were insignificant [23]. Thus, they have stated that the plants get taller with the increase of intra-row spacing. The reason behind that increase in plant heights might be the competition for light, which mostly makes the internodes thinner and taller. Not only the genetic structure of the varieties, but also the plant height and the stem diameter can be affected (increased) by close inter-row and intra-row spacing (high plant density), high doses of nitrogen fertilizers, climatic conditions, and delayed sowing dates [37,38,39,40].

Chlorophyll fluorescence serves as a direct indicator of both plant photosynthesis and the physiological condition of vegetation. In agricultural production, canopy structure plays a significant role in plant growth and development and accordingly, it influences leaf area index, SPAD unit, and maximum quantum at PSII, which are the key factors in source-to-sink association [45]. Chlorophyll fluorescence parameters are sensitive indicators of photosynthesis, and they can accurately reflect alterations in photosynthetic activity [46]. The results of this study showed a highly significant difference in terms of the maximum quantum at PSII; while in terms of SPAD values, the significant influence of the varieties was determined. Although there is limited information available on the impact of planting geometry and plant density on Fv/Fm in maize cultivars, some researchers have explored the effects of various abiotic stress factors on maize growth, yield, and yield components. Consequently, SPAD values and the maximum quantum may vary due to the genetic makeup of the varieties [47]. They demonstrated that late-maturing varieties reveal higher values of maximum quantum at PSII, while irrigation regimes [47] indicated that maximum quantum values increase with higher levels of water stress. In their study, a highly significant difference was found, with the highest Fv/Fm values observed in early maturing cultivars.

Khan et al. [41] studied two sweet corn varieties in Pakistan and found that higher plant densities led to delayed tasseling and maturity, along with decreases in kernel number and weight per ear. However, the most significant kernel yield was achieved at a plant density of 100,000 plants ha^−1^. In this study, a significant influence of both planting geometry and varieties was determined between 1.00 and 1.18. The results of this study were approximately less than the ones obtained by other researchers. These differences might be due to genetic structure and planting geometry [46,48], plant density and nitrogen doses [49,50], sowing date, and planting patterns and irrigation levels [51]. The number of kernels per ear can also vary depending on the environmental conditions and cultivation techniques, as well as the response of the kernel row number per ear and kernel number per row. The highest kernel number per ear in this study was determined in the second cropping year, 55 × 17 cm planting geometry, Khan F_1_ variety, and Challenger F_1_ 60 16 cm interaction with the mean values of 669.8, 673.9, 668.4, and 687.8, respectively. Relevant studies demonstrated different factors that influence the kernel number per ear. These factors are plant density and nitrogen dose [52], sowing date [53], and irrigation conditions [54] significantly affect the number of kernels per ear. According to Getaneh et al. [23], the differences in kernel number per ear could be attributed to widely spread plants having less plant-to-plant competition than tightly spaced plants, resulting in greater growth and more kernel numbers per ear.

Dhaliwal and Williams [55] conducted a study in the US conditions where two sweet corn varieties were planted at 10 different plant densities ranging from 42,000 to 109,000 plants ha^−1^, with an inter-row spacing of 76 cm. They observed that the marketable ear number decreased as plant densities increased, and they identified the optimum plant density to be 73,075 plants ha^−1^. Researchers revealed that marketable ear number per hectare is sensitive to environmental conditions and differences in cultivation techniques such as plant density [56], nitrogen dose [56], irrigation [54], and [57] all determined a significant difference according to each study.

The use of light, water, and nutrient resources that affect the development and yield of maize depends on the number of plants per unit area and the distribution of plants on the field [12,49]. In conditions where resources are not restrictive, more uniform plant distribution over narrower row spacing results in an increase in optimum plant density and yield, both by increasing the amount of photosynthetic light retained by the plant canopy and by reducing inter-plant competition for water and nutrients [15,16]. Looking at the results of this study, the highest values were from the year 2023, 55 × 17 cm planting geometry, Khan F_1_ variety, and Challenger F_1_ (60 × 16 cm) interaction. In comparison to the findings in the literature reviews, this research attracts attention due to the significant differences observed in fresh kernel yield. Relevant studies revealed that fresh kernel yields can vary significantly depending on location [58], sowing date [59], harvest date [60], plant density [61], irrigation treatments, and nitrogen and phosphorus doses [62,63]. Stansluos et al. [34], who investigated the adaptation of sweet corn varieties in 50 × 25 cm planting geometry, determined the kernel number and the fresh kernel yield of the varieties between 461.1–719.2 and 1681.5–11,855.0 kg ha^−1^, respectively, and the highest fresh kernel yield was obtained from Signet F_1_ followed by Challenger F_1_. Biswas et al. [37] studied the effect of two varieties of white corn PSC-121 and KS-510, and three planting geometries of 50 × 25 cm, 60 × 25 cm, and 70 × 25 cm in Manikganj/Bangladesh conditions. They demonstrated that the kernel yield increased with the increase of the inter-row spacing and the highest kernel yield (125.1 g plant^−1^) was obtained from 70 × 25 cm planting geometry. Tokatlidis et al. [64] conducted a study under Greek conditions to investigate the response of six maize varieties to plant density at 80 cm row spacing and 15, 30, and 50 cm intra-row spacing. They revealed that kernel yield per plant remained consistent across all three plant densities, while overall kernel yield increased with higher plant densities. Manan et al. [65], who investigated the densities of 55,550, 83,330, and 111,110 plant ha^−1^ in India conditions and at a distance of 60 cm inter-rows, reported that the kernel numbers per ear decreased at high densities. The highest kernel yield was obtained from the density of 83,330 plants ha^−1^.

Despite the research limitations concerning correlation analysis in terms of most of the yield and yield components, leaf area index (LAI) is the critical indicator plant’s photosynthetic capacity because LAI is linked to several physiological mechanisms. In this study, the LAI correlated positively and significantly, with EY, MEN, MEY, and FKY Commonly the higher LAI means high light interception that enhances photosynthesis leading to several chemical processes in which the plants convert the energy to essential components such as yield and yield components. The finding of our correlation results agrees with some research results. Lal et al. [66] proved a significant correlation between the grain yield and 1000-kernel weight (r = 0.363 **), Sharhrkhi et al. [67] determined a positive and significant correlation between the grain yield and the kernel rows per ear (r = 0.62 **), and the grain yield with the kernel number per row (r = 0.75 **). Though there were limitations of data concerning the traits of this study, they might show the relationship between the grain yield and some related traits.

## 4. Materials and Methods

This research was carried out in Erzurum with its terrestrial climate at 1853 m above sea level is located at 39°55′ and 41°61′ north latitude and east longitude, respectively in northeastern Türkiye. The experiment took place at Atatürk University Plant Production Application and Research Centre during the 2022 and 2023 years. The climatic data of the experiment location is presented in Table 4. In this study, three sweet corn varieties (Argos F_1_, Challenger F_1_, and Khan F_1_), which were recommended for Erzurum conditions [34], were used as plant material (Table 5). The research was carried out according to the factorial randomized complete block design with three replications, and a combination of 24 treatments consisting of three varieties (Table 5) and eight planting geometries (Table 6).

The planting geometries applied were selected in line with the research hypothesis to provide more uniform plant arrangement/distribution and higher plant density in narrower row spacing. Before the planting process, soil samples were taken from the research area and available N, P_2_O_5,_ and K_2_O for the plants and some other physical and chemical properties were determined (Table 7). In the plots, rows were opened at distances varying according to the planting geometries with the help of a marker, then two seeds were planted in the hills at a depth of 3–4 cm then the seeds were covered with soil. After the seedlings emerged with 3–4 leaves, thinning was conducted so that one seedling remained on each hill, and weeding was conducted manually using a hoe. A spacing of 1.0 m was left between the plots and 2.0 m between the blocks. Plots were fertilized manually at 200 kg N ha^−1^, 100 kg P_2_O_5_ ha^−1^, and 150 kg K ha^−1^ in order to minimize the limiting effects of NPK deficiency. During the fertilization process, 40% of the nitrogen, along with all the phosphorus and potassium, was uniformly applied to the plots before planting, where it was thoroughly mixed with the soil. The remaining 60% of the nitrogen was then distributed between the rows when the plants reached approximately 40 cm in height. The irrigation schedule was established by considering both the soil moisture content and the field capacity (FC), ensuring that moisture levels remain above the wilting point at around 60%. This is achieved through the uniform application of irrigation water through a drip irrigation system. Starting from the 25th day following the silking date and every three days, a 50 g kernel sample was taken randomly from each plot and dried in an oven set at 103 °C for 72 h and the moisture content was determined according to Standard ASABE [68]. In order to achieve high yield, when the kernel moisture drops to 73 ± 1% [69], one row from the sides of the plot and two from the ends of the plot was left as a side-effect, and the ears within the harvested area were harvested manually between 08–10 in the morning.

The days from sowing to the point when 50% of plants in each plot showed tassels were measured as days to tasseling, and the days to 50% silking were recorded similarly. Then the difference between days to tasseling and days to silking was considered as days to tasseling days to silking interval. Ten plants per plot were randomly selected to measure plant height, ear number per plant, and first ear height and LAI was calculated by the blow formula;

(1) $$LAI=Leaf\;area\;per\;plant\;\left(m^{2}\right)\times \left\lfloor \frac{Number\;of\;plants\;per\;harvesting\;area}{Harvesting\;area\;(m^{2})}\right\rfloor .$$

Before harvest, the number of plants per plot was counted and then converted to the number of plants per hectare. During the 50% silking date, the SPAD chlorophyll value was measured using a chlorophyll meter (Model SPAD 502, Minolta, Japan) at the bottom, middle, and tip of the leaf blade at the node where the first ear emerges between 9:00–11:00 AM [70]. On the same date, the maximum quantum efficiency of PII was measured by using a portable chlorophyll fluorescence system (Handy PEA+, Hansatech Instruments Kink’s Lynn, Norfolk, UK) by covering the ear leaf blade with light-holding clips and accustomed to the dark for 20–30 min. Then the measurements were made at the tip, middle, and bottom parts of the leaf blade and the average measurements were recorded [70]. After harvest, all the harvested ears were transported to the laboratory. All the ears per each plot were dehusked and weighed then converted to ear yield per hectare. Then all ears measuring 15 cm in length and 30 mm in diameter were counted and converted to the marketable ear number per hectare. From the marketable ear number, 10 ears were randomly selected and weighed, then the average ear weight was multiplied by the number of marketable ears per hectare and the results were considered as marketable ear yield per hectare.

Thus, the ten ears were used to count the kernel number per ear by multiplying the number of kernel rows per ear and the number of kernels per row. Concerning the fresh kernel yield, the kernels of the ten ears were removed using a handy kernel stripper and weighed then the average fresh kernels per ear were estimated the multiplied by the marketable ear number, and the result was considered as the fresh kernel yield per hectare.

All the data obtained from this research was subjected to the analysis variance based on the experimental design using RStudio statistical analysis software program Version: 2023.12.1+402 “doebioresearch” package, the differences among the means were compared according to Duncan multiple comparison tests (0.05) [71].

## 5. Conclusions

Planting geometry significantly affected leaf area index, Fv/Fm, ear number, ear yield, and fresh kernel yield. As an average of years, the 40 × 21 cm planting geometry resulted in the greatest marketable ear number, marketable ear yield, and fresh kernel yield. The marketable ear number, marketable ear yield, and fresh kernel yield increases due to changing the planting geometry from 70 × 15 cm (95,230 plants ha^−1^) to 40 × 21 cm (119,040 plants ha^−1^) were the result of an increase in the number of plants per unite area. The increases in these traits at the 40 × 21 cm planting geometry may be related to improvement in plant density tolerance of the varieties. The plant density was positively correlated with marketable ear number (r *=* 0.904 **), marketable ear yield (r = 0.853 **), and fresh kernel yield (r = 0.801 **). More crowding (124,220 plants ha^−1^) at the 35 × 23 cm planting geometry reduced the marketable ear yield and fresh kernel yield. This suggests that there is tolerance plant density for the sweet corn varieties used in this research to obtain maximum yields. The varieties had similar behaviors as a response to the planting geometries, regarding marketable ear number, marketable ear yield, and fresh kernel yield. The differences among the sweet corn varieties were significant for the studied traits, except for plant density and kernel number per ear. The highest marketable ear number, marketable ear yield, and fresh kernel yield were obtained from the variety Khan F_1_.

## Figures and Tables

**Table 1:** Variance analysis results of the effect of planting geometry on days to tasseling, days to silking, days to tasseling–silking interval, plant height, first ear height, plant density, leaf area index, stem diameter, SPAD chlorophyll value, maximum quantum efficiency of PSII (Fv/Fm), ear number per plant, ear yield, marketable ear number, marketable ear yield, kernel number per ear, and fresh kernel yield of sweet corn varieties as an average of 2022 and 2023 years.

		F *Values*															
Sources of Variation	df	DT ^1^	DS	DT-DS	PH	FEH	PD	LAI	SD	SPAD	Fv/Fm	ENP	EY	MEN	MEY	KNE	FKY
Blocks	2																
Year (Y)	1	564.09 *** ^2^	92.70 ***	121.63 ***	38.47 ***	61.49 ***	1.18	126.74 ***	36.78 ***	0.23	1.35	0.88	74.53 ***	4.84 *	152.62 ***	7.70 **	35.88 ***
Planting geometry (PG)	7	5.31 ***	2.87**	5.03 ***	0.60	1.58	99.32 ***	12.10 ***	1.57	1.10	7.68 ***	4.44 ***	16.61 ***	22.09 ***	41.51 ***	1.85	19.26 ***
Variety (V)	2	147.87 ***	76.66 ***	11.85 ***	27.21 ***	55.00 ***	0.37	9.80 ***	7.27 **	8.70 ***	4.34 *	5.35 **	16.84 ***	6.97 **	18.83 ***	1.36	21.88 ***
Y × PG	7	2.15 *	1.07	1.53	1.52	1.46	1.25	1.27	1.41	1.55	4.23 ***	0.27	0.63	0.98	0.86	2.43 *	2.43 *
Y × V	2	8.46 ***	11.43 ***	1.45	6.54 **	2.76	0.02	2.79	3.80 *	0.57	4.83 **	0.51	3.61 *	0.79	0.14	3.11 *	0.68
V × PG	14	1.53	1.45	1.36	1.42	1.48	0.33	1.13	1.09	1.46	2.17 *	1.27	2.24 *	0.37	1.64	0.90	0.49
Y × PG × V	14	1.68	1.65	0.64	1.26	1.50	0.93	0.97	0.73	1.68	1.54	0.36	1.62	0.56	1.13	0.94	0.56
Error	96																
Coefficient of variation (%)		1.47	1.40	14.19	3.92	7.40	3.76	12.54	6.58	3.96	2.82	9.80	11.94	10.70	9.23	4.40	17.05

^1^ DT: Days to tasseling, DS: Days to silking, DT-DS: Tasseling–silking interval, PH: Plant height, FEH: First ear height, PD: Plant density, LAI: Leaf area index, SD: Stem diameter, SPAD: SPAD chlorophyll value, Fv/Fm: Maximum quantum efficiency of PSII, ENP: Ear number per plant, EY: Ear yield, MEN: Marketable ear number per hectare, MEY: Marketable ear yield, KNE: Kernel number per ear, FKY: Fresh kernel yield. ^2^ F values with *, **, and *** are significant at the probability level of 0.05, 0.01, and 0.001, respectively.

**Table 2:** The effect of planting geometry on days to tasseling, days to silking, days to tasseling–silking interval, plant height, first ear height, plant density, leaf area index, stem diameter, SPAD chlorophyll value, maximum quantum efficiency of PSII (Fv/Fm), ear number per plant, ear yield, marketable ear number, marketable ear yield, kernel number per ear, and fresh kernel yield of sweet corn varieties as an average of 2022 and 2023 years.

Treatments	DT ^1^ (day)	DS (day)	DT-DS (day)	PH (cm)	FEH (cm)	PD (plant ha^−1^)	LAI	SD (mm)	SPAD (Unit)	Fv/Fm	ENP	EY (kg ha^−1^)	MEN (ha)	MEY (kg ha^−1^)	KNE	FKY (kg ha^−1^)
Year (Y)																
2022	67.7 ^a 2^	74.9 ^a^	7.2 ^b^	194.9 ^b^	53.8 ^b^	107,165	3.09 ^b^	17.7 ^b^	54.8	0.775	1.10	25,289 ^a^	93,061 ^a^	22,834 ^a^	656.3 ^b^	16,574 ^a^
2023	63.8 ^b^	73.2 ^b^	9.4 ^a^	203.0 ^a^	59.2 ^a^	10,6438	3.92 ^a^	18.9 ^a^	54.6	0.779	1.08	21,287 ^b^	89,481 ^b^	18,871 ^b^	669.8 ^a^	13,975 ^b^
Mean	65.8	74.1	8.3	199.0	56.5	106,801	3.50	18.3	54.7	0.777	1.09	23,288	91,271	20,853	663.1	15,274
Planting geometry (PG)																
35 × 23 cm	64.9 ^d^	74.0 ^abc^	9.1 ^a^	198.5	56.4	120,444 ^a^	4.15 ^a^	17.8	55.8	0.788 ^ab^	1.14 ^ab^	26,472 ^a^	103,418 ^a^	23,442 ^b^	659.3	17,685 ^b^
40 × 21 cm	65.6 ^bc^	73.4 ^c^	7.8 ^bc^	197.6	57.1	116,649 ^b^	3.79 ^b^	17.9	55.4	0.788 ^ab^	1.08 ^bcd^	26,495 ^a^	107,456 ^a^	24,887 ^a^	659.1	19,493 ^a^
45 × 19 cm	65.2 ^cd^	73.9 ^abc^	8.7 ^a^	196.4	56.5	113,330 ^c^	3.67 ^bc^	18.2	54.9	0.785 ^ab^	1.07 ^bcd^	25,626 ^a^	95,138 ^b^	23,336 ^b^	649.0	16,292 ^b^
50 × 18 cm	65.8 ^bc^	74.7 ^a^	8.9 ^a^	200.3	57.8	106,773 ^d^	3.47 ^cd^	18.3	55.2	0.791 ^a^	1.18 ^a^	23,682 ^b^	93,645 ^b^	22,032 ^c^	668.7	16,393 ^b^
55 × 17 cm	66.6 ^a^	74.1 ^abc^	7.5 ^c^	200.1	56.7	103,632 ^e^	3.42 ^cd^	18.4	54.8	0.790 ^ab^	1.04 ^cd^	22,096 ^bc^	88,350 ^bc^	18,816 ^d^	673.9	14,521 ^c^
60 × 16 cm	66.2 ^ab^	73.7 ^bc^	7.6 ^c^	200.3	54.6	101,538 ^ef^	3.26 ^de^	18.7	54.2	0.774 ^b^	1.11 ^abc^	21,567 ^cd^	85,542 ^cd^	19,259 ^d^	673.1	13,799 ^cd^
65 × 15 cm	66.0 ^ab^	74.3 ^ab^	8.3 ^abc^	199.8	58.0	99,739 ^f^	3.24 ^de^	18.5	54.3	0.758 ^c^	1.06 ^cd^	20,545 ^cd^	80,714 ^de^	17,958 ^de^	668.7	12,625 ^de^
70 × 15 cm	65.8 ^bc^	74.4 ^ab^	8.6 ^ab^	198.8	54.9	92,307 ^g^	3.01 ^e^	18.8	54.8	0.754 ^c^	1.02 ^d^	19,815 ^d^	75,903 ^e^	17,087 ^e^	652.9	11,387 ^e^
Variety (V)																
Argos F_1_	64.4 ^c^	73.4 ^b^	9.0 ^a^	196.9 ^b^	56.3 ^b^	106,416	3.48 ^b^	18.4 ^a^	55.1 ^a^	0.773 ^b^	1.05 ^b^	21,912 ^b^	88,745 ^b^	19,841 ^b^	662.2	14,307 ^b^
Challenger F_1_	65.2 ^b^	73.3 ^b^	8.0 ^b^	194.4 ^b^	52.2 ^c^	106,875	3.32 ^b^	17.9 ^b^	53.6 ^b^	0.774 ^b^	1.09 ^ab^	22,839 ^b^	89,523 ^b^	20,531 ^b^	658.7	14,213 ^b^
Khan F_1_	67.7 ^a^	75.6 ^a^	7.9 ^b^	205.6 ^a^	61.1 ^a^	107,114	3.71 ^a^	18.8 ^a^	55.3 ^a^	0.785 ^a^	1.12 ^a^	25,114 ^a^	95,545 ^a^	22,186 ^a^	668.4	17,304 ^a^
Interaction (V × PG)																
Argos F_1_ (35 × 23 cm)	63.3	73.2	9.8	192.9	58.5	120,789	3.92	17.5	53.2	0.780 ^a–e^	1.12	25,932 ^bc^	98,168	22,076	663.0	16,669
Argos F_1_ (40 × 21 cm)	64.2	73.0	8.8	191.7	53.6	115,489	3.77	17.8	56.7	0.765 ^b–g^	1.03	23,171 ^c–g^	105,569	22,960	652.2	18,057
Argos F_1_ (45 × 19 cm)	63.7	73.0	9.3	197.0	57.4	113,983	3.73	18.3	55.4	0.783 ^a–d^	1.00	24,112 ^cde^	93,415	23,039	648.0	15,349
Argos F_1_ (50 × 18 cm)	64.5	74.0	9.5	200.0	56.9	105,710	3.51	18.7	55.3	0.789 ^ab^	1.18	21,531 ^d–h^	95,278	20,743	672.1	16,412
Argos F_1_ (55 × 17 cm)	65.0	73.2	8.2	194.4	54.3	102,920	3.12	18.4	54.1	0.777 ^a–f^	1.00	20,036 ^fgh^	86,298	18,717	662.5	14,343
Argos F_1_ (60 × 16 cm)	65.3	73.3	8.0	198.3	54.2	101,236	3.41	18.2	54.3	0.790 ^ab^	1.07	21,484 ^d–h^	81,041	17,563	666.2	11,878
Argos F_1_ (65 × 15 cm)	64.2	72.8	8.7	202.2	59.9	99,610	3.19	18.5	55.8	0.749 ^fg^	1.00	20,152 ^fgh^	77,211	17,363	676.4	11,781
Argos F_1_ (70 × 15 cm)	65.0	74.5	9.5	198.5	55.3	91,589	3.17	19.7	56.0	0.752 ^efg^	1.00	18,875 ^h^	72,979	16,264	657.1	9965
Challenger F_1_ (35 × 23 cm)	64.3	73.2	8.8	194.1	49.9	118,473	3.90	17.1	53.5	0.790 ^ab^	1.17	27,807 ^ab^	105,335	24,743	652.3	17,174
Challenger F_1_ (40 × 21 cm)	65.3	72.3	7.0	195.8	56.2	117,063	3.57	17.7	54.7	0.777 ^a–f^	1.13	26,526 ^abc^	104,325	25,281	653.0	17,991
Challenger F_1_ (45 × 19 cm)	65.5	72.8	7.3	186.9	50.3	113,415	3.30	18.0	53.0	0.772 ^a–f^	1.10	23,635 ^c–f^	91,285	21,918	627.4	14,767
Challenger F_1_ (50 × 18 cm)	64.8	73.8	9.0	196.9	54.5	107,613	3.28	17.8	53.1	0.800 ^a^	1.20	21538 ^d–h^	91,790	21,003	657.7	15,426
Challenger F_1_ (55 × 17 cm)	66.3	73.8	7.5	198.8	53.1	103,874	3.34	18.5	55.0	0.798 ^a^	1.00	22,004 ^d–h^	85,576	17,085	677.9	13,618
Challenger F_1_ (60 × 16 cm)	65.0	72.5	7.5	199.7	51.7	102,023	3.15	18.3	53.8	0.741 ^g^	1.13	21,331 ^d–h^	85,542	19,895	687.8	13,072
Challenger F_1_ (65 × 15 cm)	65.7	74.0	8.3	190.3	51.7	100,301	3.25	18.1	52.1	0.759 ^c–g^	1.00	19,665 ^gh^	77,692	17,359	666.8	11,273
Challenger F_1_ (70 × 15 cm)	64.8	73.5	8.7	193.1	49.9	92,235	2.76	17.4	53.8	0.756 ^c–g^	1.00	20,207 ^fgh^	74,636	16,961	646.6	10,382
Khan F_1_ (35 × 23 cm)	67.0	75.7	8.7	208.6	61.0	122,072	4.64	18.7	54.8	0.794 ^ab^	1.15	25,678 ^bc^	106,751	23,508	662.5	19,212
Khan F_1_ (40 × 21 cm)	67.3	74.8	7.5	205.5	61.4	117,394	4.03	18.4	54.7	0.793 ^ab^	1.07	29,788 ^a^	112,474	26,420	672.2	22,432
Khan F_1_ (45 × 19 cm)	66.5	76.0	9.5	205.3	61.7	112,592	3.99	18.3	56.4	0.800 ^a^	1.12	29,131 ^ab^	100,715	25,052	671.6	18,759
Khan F_1_ (50 × 18 cm)	68.2	76.3	8.2	204.1	62.0	106,996	3.63	18.5	57.2	0.784 ^abc^	1.15	27,977 ^ab^	93,868	24,351	676.4	17,341
Khan F_1_ (55 × 17 cm)	68.3	75.2	6.8	207.0	62.9	104,103	3.79	18.3	55.2	0.795 ^ab^	1.13	24,248 ^cd^	93,176	20,647	681.2	15,602
Khan F_1_ (60 × 16 cm)	68.2	75.3	7.2	203.0	57.8	101,355	3.23	19.7	54.4	0.793 ^ab^	1.12	21,894 ^d–h^	90,043	20,319	665.2	16,446
Khan F_1_ (65 × 15 cm)	68.2	76.2	8.0	206.8	62.5	99,305	3.29	19.1	55.1	0.765 ^b–g^	1.18	21,832 ^d–h^	87,238	19,153	663.0	14,822
Khan F_1_ (70 × 15 cm)	67.7	75.2	7.5	204.9	59.5	93,098	3.12	19.3	54.8	0.754 ^d–g^	1.05	20,363 ^e–h^	80,095	18,037	655.0	13,816

^1^ DT: Days to tasselling, DS: Days to silking, DT-DS: Tasseling–silking interval, PH: Plant height, FEH: First ear height, PD: Plant density, LAI: Leaf area index, SD: Stem diameter, SPAD: SPAD chlorophyll value, MPSII: Maximum quantum efficiency of PSII, ENP: Ear number per plant, EY: Ear yield, MEN: Marketable ear number per hectare, MEY: Marketable ear yield, KNE: Kernel number per ear, FKY: Fresh kernel yield, Y1: 2022: Y2: 2023. ^2^ Means with the same letters are not statistically different from each other.

**Table 3:** The correlation coefficient results of the plant density, leaf area index, ear number per plant, marketable ear number per hectare, marketable ear yield, kernel number per ear, and the fresh kernel yield average of the combined data for 2022 and 2023 years.

	PD ^1^	LAI	ENP	EY	MEN	MEY	KNE	FKY
PD	1							
LAI	0.832 ** ^2^	1						
ENP	0.380	0.373	1					
EY	0.784 **	0.775 **	0.435 *	1				
MEN	0.904 **	0.828 **	0.498 *	0.845 **	1			
MEY	0.853 **	0.748 **	0.501 *	0.920 **	0.929 **	1		
KNE	−0.166	0.102	0.137	0.049	−0.038	−0.095	1	
FKY	0.801 **	0.782 **	0.496 *	0.847 **	0.957 **	0.910 **	0.055	1

^1^ PD: Plant density, LAI: Leaf area index, ENP: Ear number per plant, EY: Ear yield, MEN: Marketable ear number, MEY: Marketable ear yield, KNE: Kernel number per ear, and FKY: Fresh kernel yield. ^2^ **, * Correlation is significant at the 0.01 level and 0.05 levels, respectively.

**Table 4:** Some climate data of Erzurum province for the years 2022 and 2023 and the long-term average (LTA: “2003–2021”) ^1^.

	Total Precipitation (mm)			Average Temperature (°C)			Average Relative Humidity (%)			Minimum Temperature (°C)		Maximum Temperature (°C)	
Years	2022	2023	LTA	2022	2023	LTA	2022	2023	LTA	2022	2023	2022	2023
May	89.3	97.0	68.4	9.1	10.4	11.0	67.3	64.3	64.6	2.1	−4.1	15.9	22.5
June	80.4	63.0	42.9	15.9	14.8	15.0	62.8	66.5	59.4	7.5	2.0	23.9	25.9
July	5.2	55.6	22.2	19.4	18.2	19.3	48.1	57.0	52.7	9.5	4.0	28.4	31.8
August	0.0	4.4	16.1	21.9	21.3	19.6	36.2	42.1	49.1	11.6	6.4	31.5	36.2
September	8.8	3.3	21.3	15.5	16.4	14.2	42.6	46.3	51.8	4.3	1.9	25.7	31.0
Tot./Avg.	183.7	223.3	170.9	16.4	16.2	15.7	51.4	55.2	55.5				

^1^ It was taken from the annual climate observations of the Erzurum Regional Directorate of Meteorology.

**Table 5:** Some information about the sweet corn varieties used in this research.

Variety	Institution	Characteristic
Argos F_1_	Semillas Fito Tarım	Super sweet, maturity period 80–90 days, ear length 23–25 cm, kernel color golden yellow, tolerant to transportation
Challenger F_1_	BAYER-Seminis	Super sweet, maturity period 80–85 days, high sugar content, kernel color yellow, plant height 170–180 cm
Khan F_1_	May Seed	Super sweet, early (maturity period 76–80 days), plant height 190–200 cm, ear length 22–23 cm, ear diameter 5–5.2 cm, row number per ear 16–18, ear weight 340–350 g, kernel color dark yellow, tolerant to lodging, tolerant to transportation

**Table 6:** Planting geometries applied in the research, target plant density according to planting geometries, and plot harvest areas.

No	Planting Geometry (Inter-Row Spacing × Intra-Row Spacing)	Targeted Plant Density (plant ha^−1^)
1.	35 cm × 23 cm	124,220
2.	40 cm × 21 cm	119,040
3.	45 cm × 19 cm	116,950
4.	50 cm × 18 cm	111,110
5.	55 cm × 17 cm	106,950
6.	60 cm × 16 cm	104,160
7.	65 cm × 15 cm	102,560
8.	70 cm × 15 cm	95,230

**Table 7:** Some physical and chemical properties of the soils of the research area for the years 2022 and 2023 ^1^.

Years	Texture Class	EC (ds/m)	pH	Lime CO_3_ (%)	Available P_2_O_5_ (kg ha^−1^)	Available K_2_O (kg ha^−1^)	Organic Matter (%)	Total N (%)
2022	clay-Loam	0.18	8.0	1.04	231.0	823.7	1.50	0.18
2023	clay-Loam	0.25	7.5	1.07	203.6	910.5	1.31	0.16

^1^ Soil analyses were carried out in the laboratories of Atatürk University, Faculty of Agriculture, Department of Soils Sciences and Plant Nutrition.

## Data Availability

All data supporting the conclusions of this article are included in this article.
